# Analysis of hospitalizations due to intussusception in Sicily in the pre-rotavirus vaccination era (2003–2012)

**DOI:** 10.1186/s13052-015-0160-4

**Published:** 2015-08-01

**Authors:** Claudio Costantino, Vincenzo Restivo, Mario Cuccia, Roberto Furnari, Emanuele Amodio, Francesco Vitale

**Affiliations:** Department of Science for Health Promotion and Mother to Child Care “G. D’Alessandro” –, University of Palermo, Via del Vespro n 133, ZIP code 90127 Palermo, Italy; Infection diseases epidemiology and prevention section –, Catania Local Health Unit, Catania, Italy; Medical Doctor, Hygiene and Preventive Medicine specialist -, Catania, Italy

**Keywords:** Intestinal invagination, International classification of diseases, Hospitalization rates, Rotavirus gastroenteritis, Seasonality

## Abstract

**Background:**

Intussusception is the most common cause of bowel obstruction in infants with an incidence ranging from 9–328 cases per 100,000 infants aged 0–11 months. Causes underlining this clinical manifestation are still unknown. Possible relationship with a withdrawn tetravalent rotavirus vaccine was not confirmed by post-licensure studies and actually no increased risk of intussusception was found between infants vaccinated with both the recently licensed rotavirus vaccines. Aim of this study is to analyze the intussusception hospitalizations in Sicily from 2003 to 2012 before the introduction of rotavirus universal vaccination and its possible relation with rotavirus gastroenteritis trend.

**Methods:**

Were collected data from hospital discharge records occurred from 1^st^ January 2003 to 31^st^ December 2012 in Sicily.

Intussusception cases were defined as all hospitalizations with an ICD-9-CM code of 560.0 on any discharge diagnoses. As a proxy for the severity of cases were considered ICD-9-CM procedure codes accounting for surgical or radiologic reduction.

**Results:**

A total of 340 intussusception cases were hospitalized in Sicily from 2003 to 2012 in children aged 0–59 months. 46.8 % occurred in the age class 0–11 months.

Hospitalization rate for intussusception was 11.4 cases per 100,000 per year (32.6 cases per 100,000 among 0–11 months children; 7.3 cases per 100,000 among 12–59 months children), with a M:F sex ratio of 1.8.

During hospitalization only 25 % of intussusceptions had a spontaneous resolution, 56.5 % of cases required a surgical intervention.

From 2003 to 2012 intussusception cases were equally distributed during the year without any seasonality, while gastroenteritis hospitalizations due to rotavirus infection have a typically late winter and spring distribution.

**Conclusions:**

In Sicily from 2003 to 2012 hospitalizations due to intestinal invagination were higher among children aged 0–11 months with observed rates similar to other European countries. Regional baseline data analysis of intussusception among 0–59 children is recognized as an evidence-based public health strategy by international health authorities. Indeed, this strategy is necessary to compare any post-licensure age or sex-related change in intussusception trend after universal rotavirus vaccination introduction.

## Background

Intussusception is the invagination of one segment of the intestine within a more distal segment and it is the most common cause of bowel obstruction in infants [1].

International epidemiological data suggest that its incidence can vary greatly between different countries, ranging from 9–328 cases per 100,000 infants aged 0–11 months per year, [1] raising a question as to whether environmental factors and geographical variation may influence the development of intussusception. Although the causes underlining this clinical manifestation are still unknown, intussusception increased its popularity in the last two decades because of its potential association with rotavirus vaccination [2].

The relationship was suggested for the first time in 1998 when a tetravalent rotavirus vaccine (RotaShield) was withdrawn because of a significant excess risk of intussusception among US infants during the weeks following receipt of vaccine [3, 4]. Postlicensure surveillance studies in the United States have not confirmed the previous finding and no increased risk of intussusception was found between infants vaccinated with both the recently licensed rotavirus vaccines (RotaTeq, 2006; Rotarix, 2008) [5, 6]. More recently, in Countries with implemented Rotavirus Universal Mass Vaccination (UMV), some authors have described a small increased risk of intussusceptions for infants receiving rotavirus vaccine, although it was evident that the vaccines' benefits outweigh this risk [7–9].

To date, rotavirus vaccination represents the best strategy for reducing severe rotavirus gastroenteritis and, its associated costs due to mortality among young children and healthcare utilization. Therefore, since 2009 the World Health Organization (WHO) has recommended its introduction in all national immunization programs [10]. Despite of these recommendations, the possible association between rotavirus vaccination and increased risk of intussusception is still an argument of concern for both public health authorities, healthcare workers and general population, often representing a major limit for rotavirus vaccination [2, 7].

Moreover, WHO [11] has recently recommended that the baseline incidence of intussusception prior to the introduction of rotavirus vaccines should be based on recent local data, as temporal changes in the incidence of intussusception, unrelated to vaccination, have been reported in a number of regions [12–16]. According to this statement, the present paper has aimed to evaluate incidence rates of intussusception among infants hospitalized from 2003 to 2012 in Sicily, the first administrative Italian region that introduced rotavirus vaccination in its immunization program in January 2013.

## Methods

This study included data of hospitalizations occurred from 1^st^ January 2003 to 31^st^ December 2012 among subjects aged 0 through 59 months and resident of Sicily, a region accounting for about 5 million inhabitants with a newborn cohort of about 50,000 children per year.

Hospital discharge records (HDR) were obtained from the Sicily’s Health Regional Office, which routinely collects these data from all regional public and private hospitals. Each HDR includes demographic information (birthplace, residence, gender, and date of birth), admission and discharge dates, discharge status (categorized as “discharged/transferred” or “dead”), and up to six discharge diagnoses (1 principal and 5 secondary diagnoses) coded according to International Classification of Disease, Ninth Revision, Clinical Modification (ICD-9-CM).

Cases of intussusception were defined as all hospitalizations with an ICD-9-CM diagnosis code of 560.0 included as any of the discharge diagnoses. As a proxy for the severity of cases were considered those ICD-9-CM procedure codes which required a surgical (codes 46.80 to 46.82: intra-abdominal manipulation of the intestine, various levels) or radiologic reduction (96.29: reduction of intussusception of alimentary tract by fluoroscopy, enema, ultrasonography, water, or air). Moreover, data on rotavirus gastroenteritis (RVGE) observed in Sicily (ICD-9-CM diagnosis code of 008.61 on any diagnosis position), were also comparatively analyzed in the same period, in order to evaluate a possible link between these two pathologies [17]. Repeated cases of intussusception and/or RVGE were excluded from the analysis due to an anonymous code of identification.

The study was ethically approved by the Institutional Review Board of the Azienda Ospedaliera Universitaria Policlinico “Paolo Giaccone” of Palermo, Italy.

### Statistical analysis

Quantitative variables were expressed as mean (standard deviation = SD) when normally distributed, otherwise as median (interquartile range = IQ range). Normality was assessed using the Shapiro-Wilk test. Qualitative variables were summarized as frequency and percentage with 95 % confidence intervals (CI). Hospitalization rates (per 100,000) and CI 95 % for each investigated year were calculated using the census population for children aged five or less years [18]. Seasonality and trends in the infants’ hospitalizations were analyzed after stratifying by sex and age. To compare normally and not normally distributed variables were used Student’s *t*-test and Mann–Whitney test respectively. Chi-square and chi-square for trends tests were used to compare categorical variables. Statistical significance was set at *p* < 0.05 (p values are two-tailed). All analyses were performed using the STATA v11.2. software package.

## Results

As showed in Table 1, a total of 340 intussusception cases were hospitalized in Sicily from 2003 to 2012 in children aged 0–59 months, accounting for an average of 34 (± SD 6.4) cases per year with a mean age of 18.9 months (± SD 15.6). About half (*n* = 159; 46.8 %) of hospitalization for intussusceptions occurred in the age class 0–11 months.

**Table 1:** Characteristics of children hospitalized for intussusceptions in Sicily (2003–2012)

			*N* = 340
Gender, n (%)			
Male			218 (64.1)
Female			122 (35.8)
Age, mean in months (IC95 %)			18.9 ± 15.6
Age classes in months, n (%)			
0–11 months			159 (46.8)
12–59 months			181 (53.2)
Mean Hospitalization rate per 100,000 (IC95 %)			
0–11 months			32.6 (25.5–39.6)
12–59 months			7.3 (5.7–8.9)
Sex ratio, M:F			1.8
Mean of intussusception hospitalizations per year (± SD)			34.0 ± 6.4
Hospitalization length, median in days (IQ range)			5 (3–7)
Treatment	n (%)	Hospitalization length, median in days (IQ range)	Age, mean in months (± SD)
- Barium/air enema	63 (18.5)	4 (3–6)	20.6 (±14.8)
- Surgery	166 (48.8)	6 (4–8)	18.2 (±14.7)
- Surgery with resection	15 (4.4)	9 (8–10)	21.3 (±21.5)
- Spontaneous resolution	85 (25.0)	2 (1–4)	18.6 (±16.0)
- Unspecified surgery	11 (3.2)	7 (6–10)	21.8 (±20.7)
p-value		<0.001	0.01

Overall, during the study period the rate of hospitalization for intussusception was 11.4 (± SD 2.4) cases per 100,000 per year, greater in children aged from 0–11 months (32.6 cases per 100,000; CI 95 %: 25.5-39.6) than in children aged from 12 to 59 months (7.3 per 100,000; CI 95 %: 5.7-8.9) (*p* < 0.001).

The median length of hospitalization was 5 days (IQ range 3–7) with a M:F sex ratio of 1.8.

During hospitalization 25.0 % of intussusceptions had a spontaneous resolution, whereas, 63 cases (18.5 %) were resolved with a barium or air enema treatment and 192 (56.5 %) required a surgical intervention (15 with resection and 11 with unspecified surgery).

Length of hospital stay was longer in children with at least one surgical treatment (with or without resection or unspecified) than in children treated with air/barium enema or spontaneous resolution (median days = 6 vs. 3; *p* < 0.001). However, no statistically significant difference was found in mean age among children with different treatments (Table 1).

**Fig. 1:**
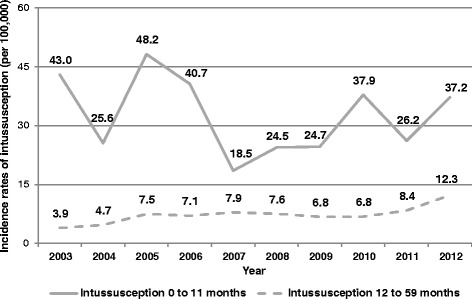
Hospitalization rates for intussusception in Sicily from 2003 to 2012

Figure 1 shows the trends of hospitalization for intussusception in Sicily that occurred, in the study period, in children aged 0–11 months and 12–59 months.

Hospitalization rates among children aged 0–11 months were characterized by a roughly wave pattern with a peak in 2005 (48.2 per 100,000). On the other hand, children aged 12–59 months reported a steadily increasing trend by time with a nearly four-fold increased risk from 2003 to 2012 (Cox-Stuart trend test: *p* < 0.05).

**Fig. 2:**
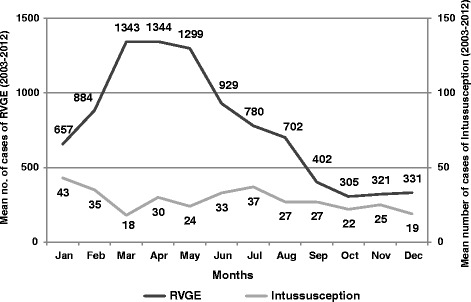
Comparison of mean no. of cases of intussusception and RVGE hospitalization seasonality in Sicily from 2003 to 2012

As shown in Fig. 2, in Sicily from 2003 to 2012 intussusception cases were equally distributed during all over the year without any seasonality. On the contrary, RVGE hospitalization were observed especially during late winter and spring, with a peak in the month of April (1,344 cases) and a high number of cases also in March (1,343) and May (1,299) (Fig. 2).

**Fig. 3:**
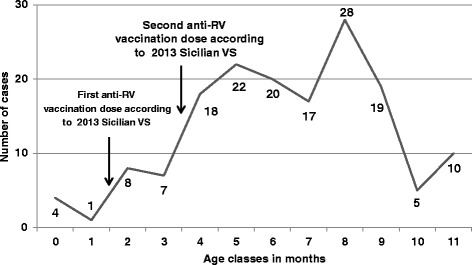
Cases of intussusception in the first year of life for age classes in months in Sicily from 2003 to 2012. In figure are indicated administration times of the first and second dose of anti-rotavirus (RV) vaccination according to Sicilia vaccination schedule (VS) of 2013

Finally, analyzing the distribution of intussusception hospitalization among children aged 0–59 months, was observed a peak at 8 months (28 cases in the 10 years of observation) (Fig. 3).

## Discussion

Intussusception is a rare clinical condition that is observed in almost all regions of the world, particularly among infants, regardless of rotavirus vaccination [1]. Considering that a large majority of intussusception cases are diagnosed and treated in hospital, post-marketing surveillance can be successfully carried out by hospital discharge databases that allow a timely and inexpensive collection of epidemiological information and include standardized diagnosis code lists that enable secular trend analysis of incident disease.

Results reported in the present study should be considered as the baseline incidence rate of hospitalization for intussusception in Sicily in the anti-rotavirus pre-vaccination era. Our results demonstrate among children aged 0–11 a number of hospitalization lower (32.6 per 100,000) than those estimated in other European countries with active surveillance systems, such as Switzerland (38.0/100,000) and Germany (60.4/100,000) [19, 20]. While underreporting can occur in active surveillance systems which rely a lot on clinical interest and involvement, underestimation is also possible in passive surveillance systems due to coding/misclassification errors or by not including study subjects treated in outpatient/short stay settings [19, 21, 22].

In line with other studies, our findings showed a male predominance in the incidence of intussusception in Sicily [15, 23]. Reasons for this finding may include changes in feeding practices affecting the infant gut, maturation of lymphoid tissue or a decline in maternal antibodies against infectious agents possibly associated with intussusception [15]. According to literature there was no possible explanation of the intussusception male predominance observed worldwide. Future study should enhance the possible effects caused by sexual hormones through specific analysis.

In the current study, length of hospital stay was not associated with age but only with kind of intervention. Indeed, children aged <12 months had a median hospitalization length similar to children aged ≥12 months. Conversely, children aged ≥12 months were least likely to require surgery, confirming data reported in previous studies [24, 25].

Frequently, in younger children the clinical expression of intussusception is absent or indefinite, therefore establishing the diagnosis based on the classic symptoms alone may cause delay and, in turn, would result in a higher rate of surgical intervention and intestinal resection [26].

Moreover, in Sicily, intussusception monthly trend could not be related with observed RVGE rates and peaks [17]. Indeed, our data confirmed the lack of seasonality of intussusceptions in accordance with other studies [19, 27]. The frequent association of intussusception with hypertrophy of Peyer patches and mesenteric lymphadenopathy raises the possibility of one or more infectious causes. In this sense, several pathogens, such as adenovirus, human herpes virus HHV-6 and HHV-7, Salmonella spp, E.coli, Campylobacter, and Shigella spp, have been found in children with intussusception [28]. Further studies may need to explore the factors underlying the risk for intussusception and its possible association with other infections. Finally, it should be pointed out that, consistently with data reported among other researchers [29], in our population the peak of intussusceptions was found at 8 months of age. Whilst, in Sicilian and other European settings [17, 30] the peak of RVGE incidence was about 12 months of age. These data seem to reinforce the lack of a causal association between RVGE and intussusception.

Our data confirm that intussusception is an extremely rare condition in children under 5 months of age and suggest that a correct administration of the anti-RV vaccination within the first 5 months of age could avoid an overlap of physiological and vaccine related intussusception.

A limit of this study can be that from a previous study found that more than 40 % of intussusception cases were managed and discharged directly from emergency department (ED) or short-stay settings and could be missed in analyses using only inpatient discharge data [5].

If the proportion of intussusception cases admitted from an ED setting is stable over time, inpatient data on intussusception should be a reasonable proxy for overall intussusception rates and should reflect trends over time.

Another limit of the study, due to restricted information included in Sicilian HDRs, is the rather weak association between RVGE and intussusception which cannot be supported with additional statistical analysis.

## Conclusions

In general, considering that the use of regional baseline data is of paramount importance when evaluating postlicensure trends of intussusceptions for assessing any potential vaccine-associated risk, the observed rates could represent a significant evidence-based public health strategy, allowing to monitor any age-related or sex-related change in intussusception risk following the introduction of the universal rotavirus vaccination among infants in Sicily.

Finally, our data demonstrated the different seasonality of intussusception and RVGE hospitalizations, hypothesizing a lack of temporal association between these pathological condition. Considering that available rotavirus vaccine was mainly constituted by live attenuated virus, any temporal association with vaccine or significant increase in intussusception rate would be expected in post-licensure studies.

## Abbreviations

RVGE: Rotavirus gastroenteritis
HDR: Hospital discharge record
UMV: Universal mass vaccination
WHO: World Health Organization
ICD-9-CM: International Classification of Disease, Ninth Revision, Clinical Modification
SD: Standard deviation
IQ range: Interquartile range
CI: Confidence interval
ED: Emergency department
